# Immunized mice naturally process *in silico*-derived peptides from the nucleocapsid of SARS-CoV-2

**DOI:** 10.1186/s12866-023-03076-5

**Published:** 2023-10-28

**Authors:** Mario Aldair Campos-Ruíz, Berenice Illades-Aguiar, Oscar Del Moral-Hernández, Mariana Romo-Castillo, Marcela Salazar-García, Mónica Espinoza-Rojo, Amalia Vences-Velázquez, Karen Cortés-Sarabia, Victor M. Luna-Pineda

**Affiliations:** 1 Laboratorio de Inmunobiología y Diagnóstico Molecular, Facultad de Ciencias Químico Biológicas, Universidad Autónoma de Guerrero, Chilpancingo de los Bravo, Guerrero, México; 2 Laboratorio de Biomedicina Molecular, Facultad de Ciencias Químico Biológicas, Universidad Autónoma de Guerrero, Chilpancingo de los Bravo, Guerrero, México; 3 Laboratorio de Virología, Facultad de Ciencias Químico Biológicas, Universidad Autónoma de Guerrero, Chilpancingo de los Bravo, Guerrero, México; 4 Laboratorio de Investigación en COVID-19, Laboratorio de Investigación en Inmunología y Proteómica, Hospital Infantil de México Federico Gómez, Ciudad de México, México; 5 Laboratorio de Biología del Desarrollo y Teratogénesis Experimental, Hospital Infantil de México Federico Gómez, Ciudad de México, México; 6 Programa Investigadoras e Investigadores por México, Consejo Nacional de Humanidades, Ciencias y Tecnologías, Ciudad de México, México

**Keywords:** SARS-CoV-2, Nucleocapsid protein, Peptides, *In silico*, Immunoassays

## Abstract

**Background:**

The nucleocapsid (N) protein of severe acute respiratory syndrome coronavirus-2 (SARS-CoV-2) is an excellent immunogen that promotes the production of high-titer antibodies. N protein-derived peptides identified using a bioinformatics approach can potentially be used to develop a new generation of vaccines or diagnostic methods for detecting SARS-CoV-2 and its variants. However, further studies must demonstrate their capacity to be naturally processed by the immune system.

**Objective:**

We aimed to examine the in vivo processing and recognition of *in silico*-identified peptides using the serum of immunized animals with the complete protein.

**Methods:**

Recombinant N (Nrec) protein was subcutaneously administered to six Balb/c mice. Enzyme-linked immunosorbent assay (ELISA), western blotting, dot blotting, and immunoprecipitation were performed to evaluate the recognition of the complete protein and *in silico-*derived peptides.

**Results:**

The serum of immunized mice recognized ~ 62.5 ng/µL of Nrec with high specificity to linear and conformational epitopes. Dot blot analysis showed that peptides Npep2 and Npep3 were the most reactive.

**Conclusion:**

Our data confirm the high immunogenicity of the SARS-CoV-2 N protein and provide evidence on the antigenicity of two peptides located in the N-arm/RNA-binding domain (Npep2) and oligomerization domain/C-tail (Npep3), considered the biologically active site of the N protein.

**Supplementary Information:**

The online version contains supplementary material available at 10.1186/s12866-023-03076-5.

## Background

Severe acute respiratory syndrome coronavirus-2 (SARS-CoV-2) is the etiological agent of the pandemic disease COVID-19. SARS-CoV-2 is an enveloped virus with positive-sense single-stranded RNA with a ~ 30 kb genome [1, 2]. SARS-CoV-2 comprises four structural proteins: spike (S), membrane (M), envelope (E), and nucleocapsid (N) [3]. N protein is the most abundant in the inner part of the virion and participates in packing RNA in the ribonucleoprotein complex [4]. Additionally, it engages in RNA replication/transcription, virion assembly, and immune response evasion by acting as an antagonist of antiviral activities during the viral cycle [5]. N protein is composed of 419 amino acids and is structurally divided into intrinsically disordered regions (N-arm and C-tail), followed by two conserved structural regions (RNA-binding and oligomerization domains), which are linked by a central Ser/Arg (SR)-rich, flexible linker region (LKR) [6].

N protein is an excellent immunogen for activating the humoral response that includes high titers of IgG, IgG1, IgG2a, and IgM-specific anti-N antibodies, which are reportedly maintained for > 5 months [7]. We have reported that anti-N protein antibodies are present in children and adolescents recovering from COVID-19 and are associated with their demographics and clinical features [8]. Nevertheless, adult patients with COVID-19 also have high levels of anti-N protein antibodies that correlate with disease severity [9]. All findings show the potential of N protein for developing a new generation of vaccines or alternative methods for detecting SARS-CoV-2 and its variants [10].

The use of bioinformatics tools has improved the search for immunogenic peptides for designing vaccines using structural proteins of pathogens, such as SARS-CoV-2 [11, 12]. Immunoinformatic analysis has been used to identify COVID-19 vaccine targets, such as the epitope SRGGSQASSRSRSRSRNSSRNSTPGSSRGTS located between amino acids 176–206 [13]. Whole proteome screening and immunoinformatic analysis of SARS-CoV-2 revealed 21 epitopes from 7 different proteins of SARS-CoV-2; the best epitopes were from S (ATSRTLSYY) and N (KAYNVTQAF) proteins [14]. Recently, we used immunoinformatic analysis to identify immunogenic and antigenic peptides in S and N proteins of SARS-CoV-2 and reported a list of peptides for both proteins [15].

N protein is a potential target because it is highly conserved, stable, and has fewer mutations than other SARS-CoV-2 proteins, such as S protein [16]. Therefore, we aimed to provide evidence on the in vivo processing and recognition of *in silico*-identified peptides using the serum of immunized animals with the complete protein.

## Methods

### Expression and purification of Nrec

Nrec was expressed and purified according to Cortés-Sarabia et al. [8]. The constructed plasmid was named pLATE-51/Nrec and was transformed into *E. coli* Rosetta cells. Nrec expression was induced for 5 h at 37 °C with 1 mM IPTG (Thermo Scientific). The recombinant protein possesses a 6× HIS tag at the N terminal that allowed its purification via metal affinity chromatography under native conditions using Hispur Ni-NTA HisPur resin (Thermo Scientific).

### Immunization of Balb/c mice

Six male Balb/c mice were subcutaneously immunized with 10 µg Nrec diluted in PBS (pH 7.0), mixed with incomplete Freund’s adjuvant (Sigma-Aldrich), in a total volume of 200 µL per animal. Three immunizations were performed on days 1, 56, and 87, and sera samples from the mice were collected on days 1, 28, 52, 77, and 127. At the end of the experiment, mice were euthanized with CO_2_. In this study, no animals were euthanized for sickness or distress, and animal management was performed according to institutional animal care guidelines. In addition, we adhered to the ARRIVE guidelines (https://arriveguidelines.org) for treatment and manipulation, and all experiments were performed under NOM-062-ZOO-1999.

### Peptide selection and synthesis

Peptides were identified by *in silico* analysis from the amino acid sequence of SARS-CoV-2 N protein (NCBI Protein Database, Access Number: QJX60131.1) and selected based on their predicted immunogenic characteristics, antigenicity, major histocompatibility complex (MHC)-binding ability, and B and T cell activation, using the B cell epitope prediction tools Bepipred-1.0 and Bepipred-2.0 (http://tools.iedb.org/bcell/) and the antigenic predictor (http://imed.med.ucm.es/Tools/antigenic.pl). The selected peptides were synthesized as multi-antigenic peptide 8 (MAP8), which allows the synthesis of peptides with a length < 15 residues. The peptides were synthesized by PepMic (http://www.pepmic.com/), according to Cortés-Sarabia et al. (2022). Each peptide was suspended in a final concentration of 1 mg/mL [15].

### Enzyme-linked immunosorbent assay (ELISA)

Indirect ELISA was used to evaluate the immune response in immunized mice, antigenic mapping, titration of antibody dilution and antigen concentration, and antibody recognition of Nrec protein. Briefly, high-binding 96-well plates (Corning) were coated with the antigen (Nrec protein or peptides in MAP8 format), diluted in carbonate buffer (50 mM Na_2_CO_3_/NaHCO_3_, pH 9.6), and incubated overnight at 4 °C. Then, 200 µL/well with 5% skimmed milk diluted in phosphate-buffered saline/Tween 20 (PBS-T) was added to the plate for blocking and incubated for 40 min at 37 °C. Next, 100 µL/well of the individual serum sample of each mouse or with the diluted pool in PBS was added to the plate and incubated for 2 h at 37 °C. Finally, the plate was incubated with mouse anti-IgG H + L coupled to horseradish peroxidase (HRP) (Invitrogen) for 2 h and washed thrice with PBS-T. The enzymatic reaction was developed using *o*-phenylenediamine dihydrochloride (Sigma-Aldrich) and stopped using 2 N H_2_SO_4_ (Sigma-Aldrich). Optical density was measured at 492 nm using a microplate reader.

### Western blotting and dot blotting

For western blotting, 20 µg of the total lysate of *E. coli* Rosetta cells overexpressing Nrec was mixed with 1× Laemmli loading buffer for denaturation at 98 °C for 8 min. Protein separation was performed using 12% SDS-PAGE. Separated proteins were transferred to a polivinildenedifloride membrane (Merck). The membranes were blocked with 5% skimmed milk diluted in PBS-T for 40 min with constant agitation at room temperature (RT) for 2 h. Subsequently, the membranes were incubated with the primary antibody (pool or serum samples), followed by mouse anti-IgG H + L antibody coupled to HRP (Invitrogen) diluted at 1:2,000 and 1:6,000, respectively, and incubated for 2 h at RT with agitation. Between each step, the membranes were washed thrice with PBS-T. Finally, the enzymatic reaction was developed using diaminobenzidine and hydrogen peroxide diluted in PBS (pH 7.2). The reaction was stopped with distilled water. Dot blotting was performed similarly, but Nrec protein and peptides in MAP8 format were immobilized on nitrocellulose membranes directly. Blot images were analyzed with the ImageJ image processing program version 1.53t (National Institutes of Health, Bethesda, MD). The Ii value was defined as the sum of pixel intensity over all pixels in an object.

### Immunoprecipitation

For immunoprecipitation, 5 µL of serum was mixed with 15 µL of A/G-coupled to agarose beads (Santa Cruz Biotechnology) and 1.2 mL of PBS for 2 h at RT with constant agitation and centrifuged at 2,898 × *g* for 5 min. The pellet was washed thrice with 1 mL PBS (pH 7.2). Then, 10 µL lysate of *E. coli* Rosetta cells overexpressing Nrec and 1.2 mL of PBS were incubated with the bead–antibody complex for 2 h at RT, with constant agitation. Finally, the complexes were washed and used for 10% SDS-PAGE. The gels were stained with Coomassie blue (BioRad).

## Results

**Fig. 1:**
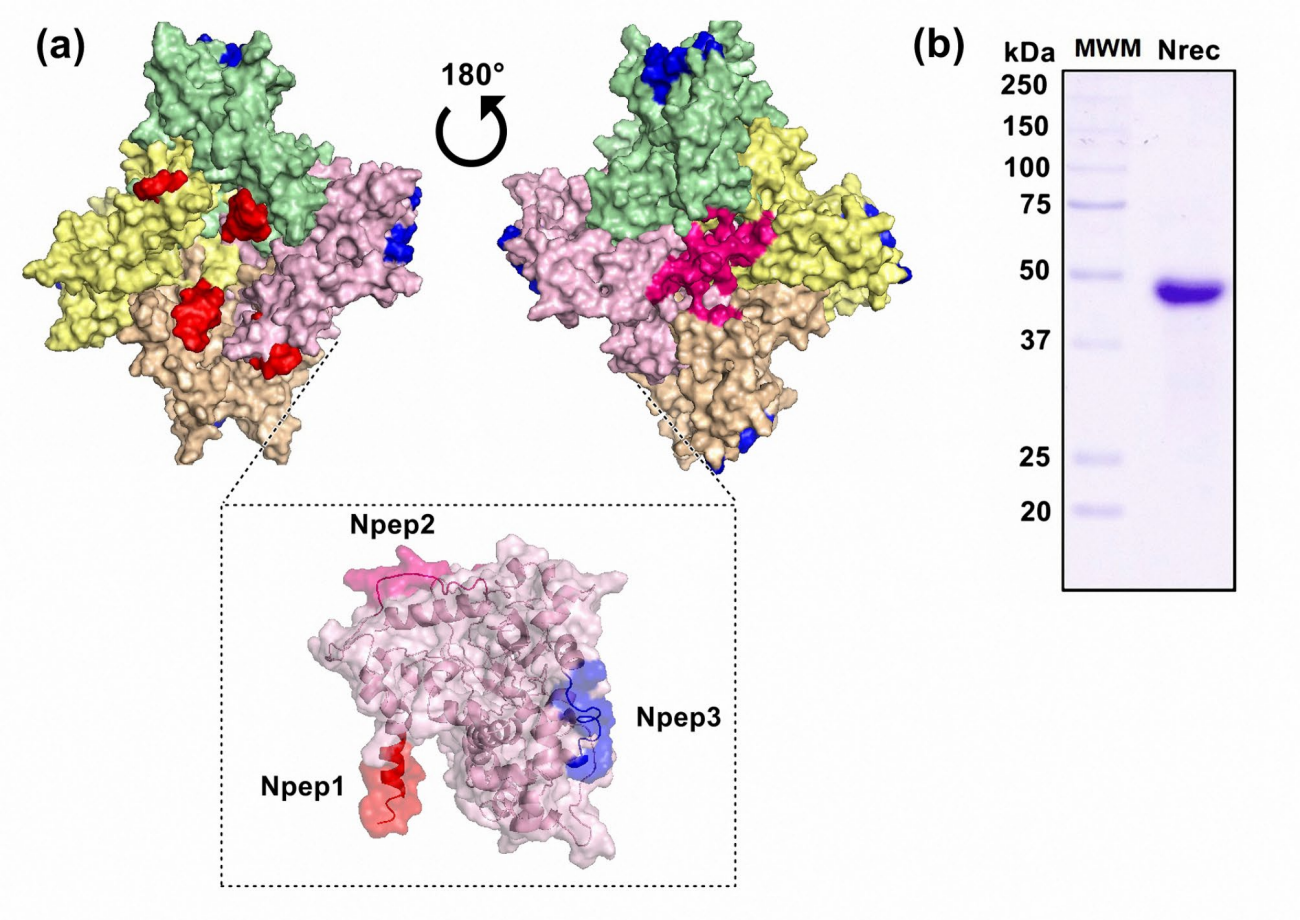
Structure of the nucleocapsid protein: localization of selected peptides and purification. (**a**) 3D structure of Nrec. The tetramer of N protein shows each monomer in a different color. Selected peptides are represented as Npep1 (red), Npep2 (pink), and Npep3 (blue). (**b**) Purification of Nrec. 12% SDS-PAGE stained with Coomassie blue. MWM: Molecular weight marker dual color (BioRad). The gel was edited using clear delineation with dividing lines. The full-length gel is included in Fig S1 [see Supplementary Information file 1]

Nrec contains 448 amino acids and has a theoretical molecular weight of 48.7 kDa. Structurally, Nrec comprises four monomers (Fig. 1a). Nrec is reportedly soluble, with purity > 90% (Fig. 1b). Based on our study [7], three peptides were selected from the immunoinformatic analysis. The selection criteria included their predicted immunogenic characteristics, antigenicity, MHC binding capacity, and B and T cell activation [15]. The peptides are shown in Fig. 1a, and their features are listed in Table 1.

**Table 1:** Features of N protein peptides

Peptide	Length (Amino acids)	Sequence	Localization
Npep1	12	MSDNGPQNQRNA	N-terminal (N-arm)
Npep2	9	RSKQRRPQGLPN	N-arm/RNA-binding domain
Npep3	7	DAYKTFPPTEPK	Oligomerization domain/C-terminal (C-tail)

### Nrec is immunogenic

**Fig. 2:**
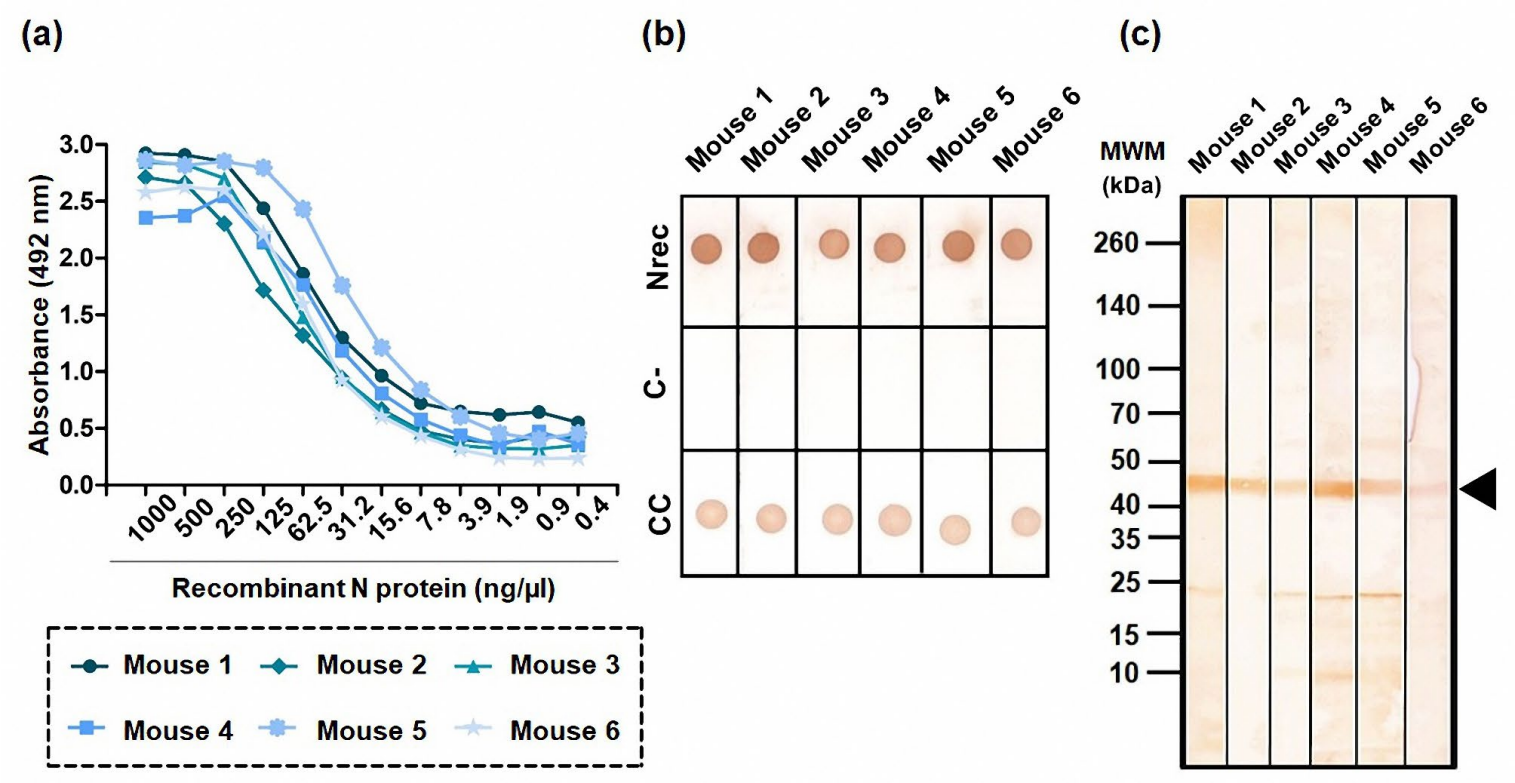
Immunogenicity of Nrec in immunized mice. (**a**) Indirect ELISA. Nrec (1,000–62.5 ng/µL) was used as antigen. (**b**) Dot blotting. The antigen was fixed to a final concentration of 1 µg. (**c**) Western blotting. MWM: Molecular weight marker. Black arrow indicates Nrec. In all experiments, we used serum samples derived from immunized mice as the primary antibody and mice anti-IgG coupled to HRP (1:10,000) as the secondary antibody. The blots were edited using clear delineation with dividing lines. Full-length blots are included in Fig S2 [see Supplementary Information file 1]

Nrec was used to immunize six Balb/c mice, and the immune response was evaluated five times, one before immunization and four after the first immunization. From the third post-baseline sample (day 77), high levels of antibody titers were observed at 1:8,000 in all animals [see Additional file 1]. Nevertheless, the serum sample of the fourth evaluation was used for the following experiments. The serum sample of each mouse was characterized by indirect ELISA, dot blotting, and western blotting to confirm recognition of the complete protein (Fig. 2). Indirect ELISA showed that reactivity of Nrec ranged from 1,000 ng/µL to 62.5 ng/µL (Fig. 2a). Dot blotting provided evidence on recognizing native Nrec (Fig. 2b), and western blotting identified the denatured antigen (Fig. 2c). Western blot analysis showed that the anti-Nrec antibodies were highly specific when evaluated with the total bacterial extract.

**Fig. 3:**
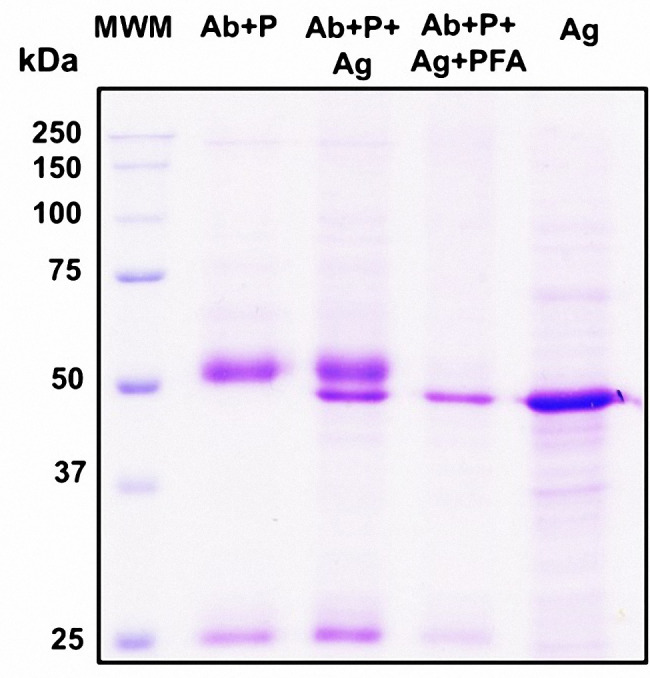
Immunoprecipitation of Nrec. 10% SDS-PAGE gel stained with Coomassie blue. Lane 1: MWM (molecular weight marker). Lane 2: Ab + P (antibody + A/G agarose beads). Lane 3: Ab + P + Ag (antibody + A/G agarose beads + antigen Nrec). Lane 4: Ab + P + Ag + PFA (antibody + A/G agarose beads + antigen Nrec + 3% paraformaldehyde). Lane 5: antigen (total lysate of Rosetta strain of *Escherichia coli* transformed with pLATE-51/Nrec). The gel was edited using clear delineation with dividing lines. The full-length gel is included in Fig S3 [see Supplementary Information file 1]

As a complementary analysis, we mixed the sera of the six mice to corroborate the specific recognition of Nrec by immunoprecipitation from the total lysate of *E. coli* Rosetta cells (Fig. 3). All findings confirmed that SARS-CoV-2 N protein is a good immunogen.

### The immune system naturally processes *in silico*-identified peptides derived from N protein

**Fig. 4:**
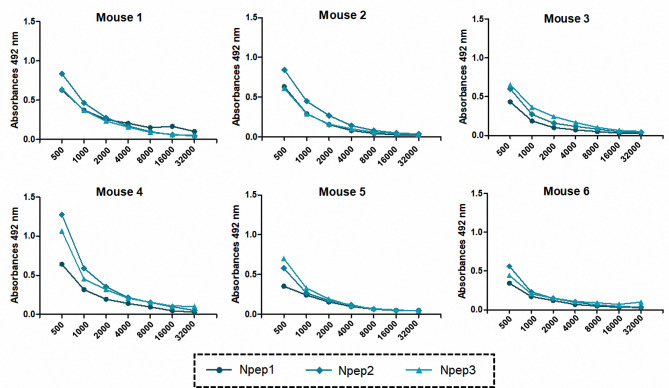
Indirect ELISA to test the recognition of the peptides by the immunized mice. We used the three selected peptides (Npep1, Npep2, and Npep3) as antigens at a final concentration of 1 µg/mL. The primary antibody (serum sample) was diluted from 1:500 to 1:32,000. Anti-mice IgG coupled to HRP (1:10,000) was used as the secondary antibody

We evaluated the antigenicity of the *in silico*-identified peptides and examined their ability to be naturally processed by antigen-presenting cells, as well as their coupling to MHC to induce the humoral response (Table 1). We used serum samples from the six immunized mice and observed that Npep2 and Npep3 were the most reactive antigens. The serum of mouse 4 presented the highest recognition of Npep2, followed by Npep3 (Fig. 4).

**Fig. 5:**
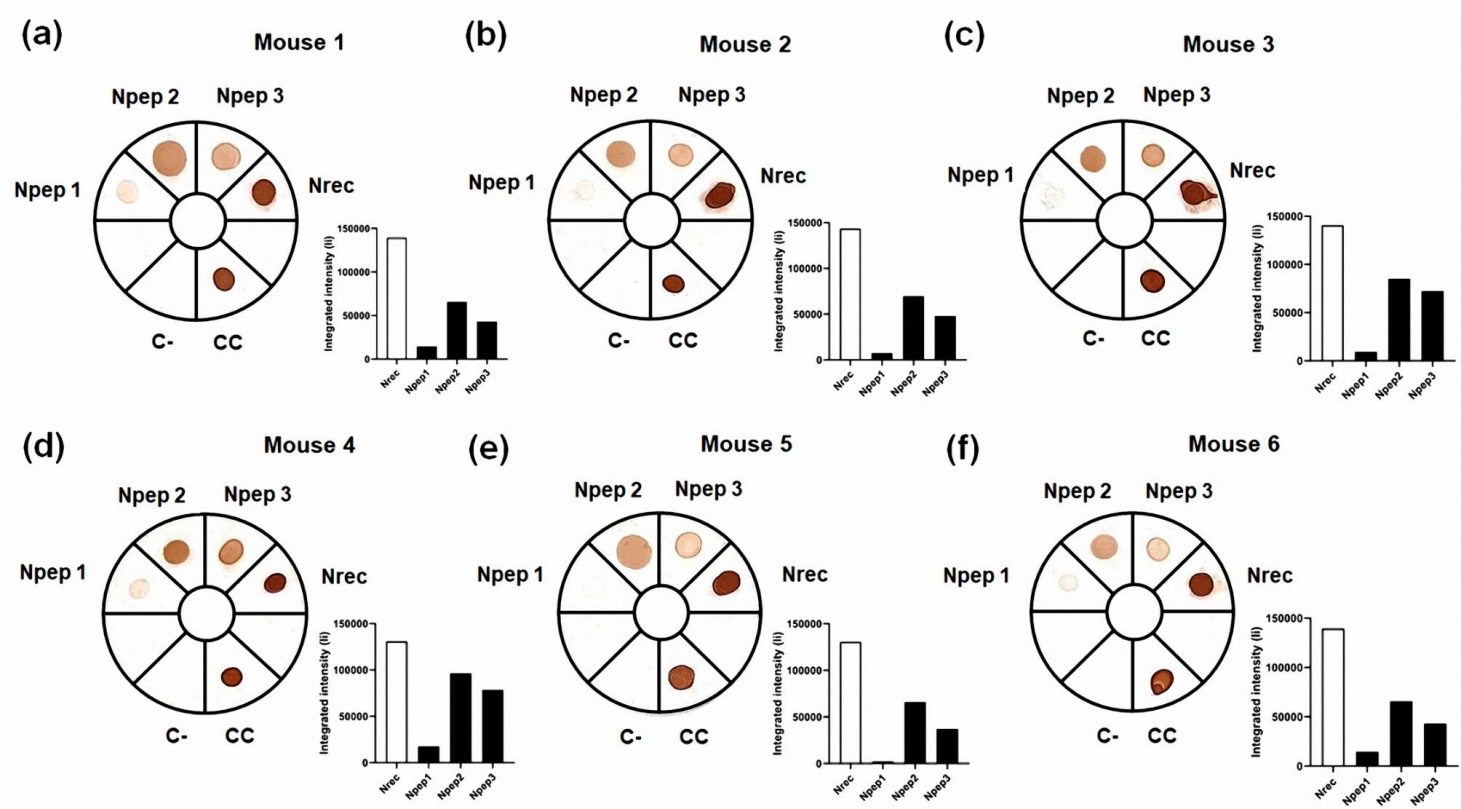
Dot blotting to test peptide recognition by immunized mice. One microgram of Nrec and peptides was fixed on nitrocellulose membranes. Primary antibody: serum samples (1:2,000). CC: conjugate control. Graphs represent integrated intensity values of each blot: white bar represents Nrec, and black bars represent the three peptides. The blots were edited using clear delineation with dividing lines. Full-length blots are included in Fig S4 [see Supplementary Information file 1]

To confirm that the serum samples of mice could recognize *in silico*-identified peptides, we performed dot blotting followed by the densitometric analysis of the dots. Sera from all mice showed high integrated intensity (Ii) values between 12.7 × 10^4^ (mouse 1) and 14.3 × 10^4^ (mouse 2). Npep2 had the highest Ii values between 65.8 × 10^3^ (mouse 6) and 96.3 × 10^3^ (mouse 4) when compared with other peptides (Fig. 5). Thus, Npep2 and Npep3 are highly antigenic compared with Npep1, which had low Ii values.

## Discussion

SARS-CoV-2 is the main etiological agent of COVID-19. SARS-CoV-2 comprises four structural proteins, of which S and N are the most studied. S protein allows interaction with the host receptor angiotensin-converting enzyme 2, and N protein is associated with the ribonucleoprotein complex inside the virion [17]. S and N proteins are the major immunogenic proteins detected during SARS-CoV-2 infection [18]. Although S protein has been widely used in developing techniques for virus detection, N protein is more conserved, has fewer mutations than S protein, and has immunogenic and antigenic characteristics [19, 20]. Recent studies have proposed the use of N protein as a biomarker to detect SARS-CoV-2 in serum samples at the onset of symptoms, reducing the probability of developing severe symptomatic disease and spreading the virus to healthy individuals [21]. N protein could be essential for the host’s immune response and early diagnosis of the disease; therefore, analyzing the complete protein and derived peptides is a new insight for developing diagnostic methods or antibodies [22]. In this study, we report the high efficiency of N protein to produce specific antibodies using a mouse model.

N protein of SARS-CoV-2 is closely related to the N proteins of SARS-CoV (90%) and MERS-CoV (50%) and can promote cross-reactivity [23]. Bioinformatics studies allow us to select specific SARS-CoV-2 N-peptides that can bind to MHC-II. Using peptides has several advantages compared with recombinant proteins, such as better stability, less production cost, and the ability to be chemically synthesized [24]. Studies have reported the presence of peptides at the C-arm region, LKR domain (LKR peptides), and C-tail region (CTD peptides) of the N protein. Antibodies against CTD peptides were reported in patients with intubation, whereas antibodies against NTD and LKR peptides were related to severe COVID-19 [25]. Our analysis suggests that Npep2 (RSKQRRPQGLPN) was the most antigenic. This peptide is localized between the N-arm and RNA-binding domain of the N protein, a region that has not been reported. The RNA-binding domain structure is like an overall right-hand-like fold composed of a β-sheet core with an extended central loop [4]. The core region adopts a five-stranded U-shaped right-handed antiparallel β-sheet platform with a β4–β2–β3–β1–β5 topology, flanked by two short α-helices (α1 before β2 strand; α2 after β5 strand) [4]. Npep2 is localized at the end of the N-arm domain near the β1 sheet and conformationally near β1–β5 (Fig. 1). Peptides in the RNA-binding domain are reactive during the early recovery of COVID-19, whereas CTD peptides are more reactive during late recovery [26]. These findings indicate that Npep2 can be used as a biomarker to identify severe cases and prevent complications.

Several computational approaches as predictive algorithms of epitopes have been used to design multi-epitope vaccines, such as cytotoxic T lymphocyte, helper T lymphocyte, and linear B lymphocyte epitopes [27, 28]. Thus, bioinformatic tools can be used to evaluate the peptides’ allergenicity and toxicity, as well as their ability to induce IFN-γ production (helper T lymphocytes) [27, 29]. In addition, bioinformatic tools can also be used for monitoring the genetic variations that permit swift adapt vaccine formulations to maintain their effectiveness [30].

Previous immunoinformatic analysis identified peptides to develop COVID-19 vaccines. The peptide SRGGSQASSRSRSRSRNSSRNSTPGSSRGTS is in the RNA domain/LKR, whereas peptide KAYNVTQ is in the oligomerization domain and the peptide MSDNGPQNQRNAPRIT is situated between the oligomerization domain and C-tail [13]. These peptides were considered candidates for vaccine formulation; however, neither is localized between the C-arm and RNA-binding domains.

Notably, peptide-based vaccines using N protein can identify SARS-CoV-2 regardless of the variant, such as delta and omicron, because N protein has a lower mutation rate than S protein [31]. Another critical consideration is the impact of N-based vaccines on other diseases. Recently, the SARS-CoV-2 N protein was reported to share significant molecular overlap with multiple sclerosis-associated proteins, including myelin proteolipid protein [32]. In addition, T-cell-activated N protein peptides could help understand neuronal pathological sequelae of SARS-CoV-2.

The main reason to identify and provide scientific evidence on the biological relevance of N-derived *in silico*-identified peptides is their potential utility as diagnostic, protective, and treatment tools to prevent severe cases of COVID-19 and to perform research on other diseases, such as multiple sclerosis.

Some limitations of this study should be considered. Although the bioinformatics tool has been extensively used in immunology, selecting parameters and algorithms has limitations. In addition, this study included only the major antigenic/immunogenic peptides from N protein. We determined the antigenicity of peptides chosen from the N protein; nevertheless, the peptides must be individually administered in animal models to assess the immunogenicity.

## Conclusions

Our data confirm the high immunogenicity of Nrec protein derived from SARS-CoV-2 to generate highly specific antibodies. We provide evidence on the antigenicity of peptides Npep2 (N-arm and RNA-binding domain) and Npep3 (oligomerization domain and C-tail) located in biologically active sites of the N protein. Further studies must test the immunogenicity of the peptides by individual administration in animal models. The findings would form a basis for the development of a SARS-CoV-2 vaccine or the production of monoclonal antibodies to detect the native protein in biological samples. Our data provide insights into testing the antigenicity of *in silico*-identified peptides.

### Electronic supplementary material

Below is the link to the electronic supplementary material.

Supplementary Material 1

Supplementary Material 2

## Data Availability

All data supporting this study’s findings are available from the corresponding authors (K.C. and V.L.) upon reasonable request.

## List of abbreviations

SARS-CoV-2: severe acute respiratory syndrome coronavirus-2
N: nucleocapsid
Nrec: recombinant nucleocapsid
Aa: amino acids
LKR: central Ser/Arg(SR)-rich flexible linker region
MAP8: multi antigenic peptide 8
PBS-T: phosphate-buffered saline/Tween 20
Ii: Integrated intensity
