# Supplementary material for: Immunized mice naturally process in silico-derived peptides from the nucleocapsid of SARS-CoV-2
Source: BMC Microbiol. 2023 Oct 28;23:319. doi: 10.1186/s12866-023-03076-5 (PMC10612231; doi:10.1186/s12866-023-03076-5)
Supplement: Supplementary file 1 — Supplementary Material 1 [file 12866_2023_3076_MOESM1_ESM.pdf]

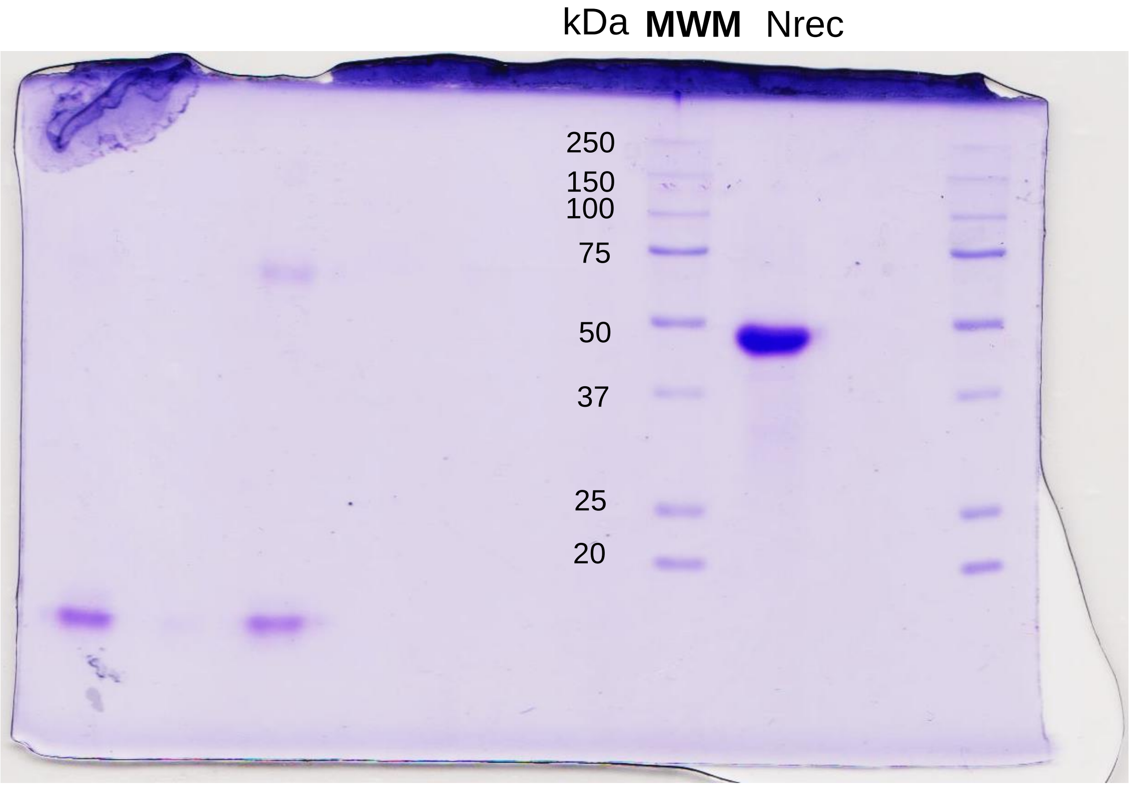

Fig S1. Structure of Nucleocapsid protein: localization of selected peptides and purification.

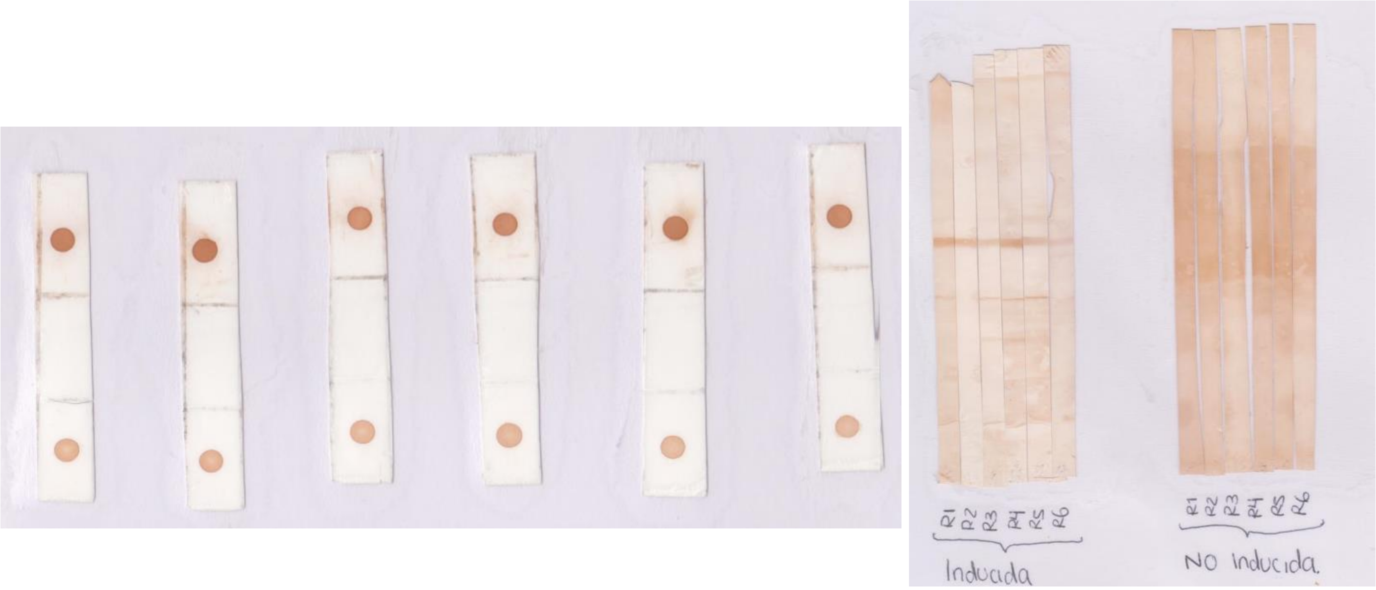

Fig S2. Immunogenicity of Nrec in immunized mice.

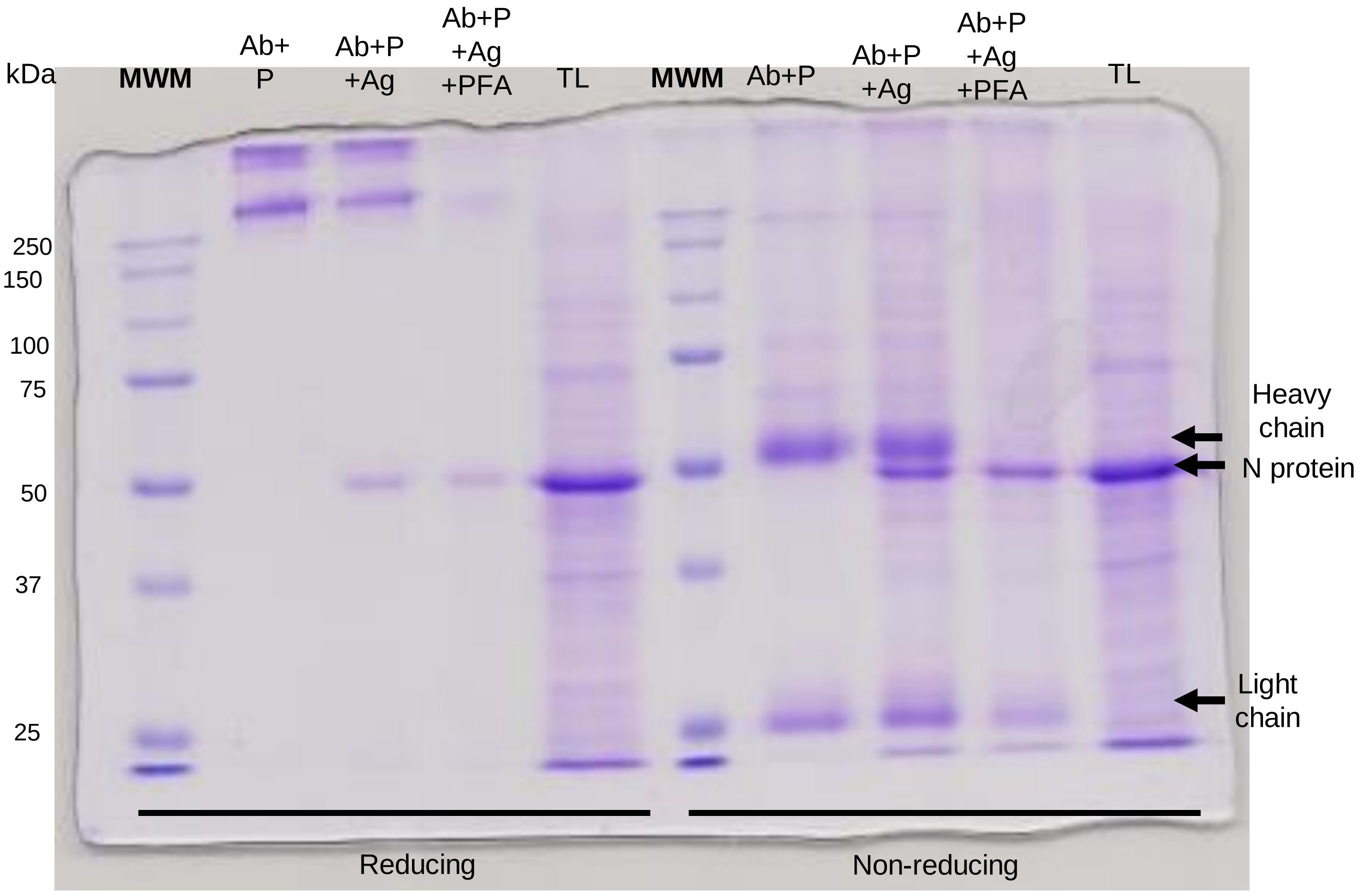

Fig S3. Immunoprecipitation of Nrec protein.

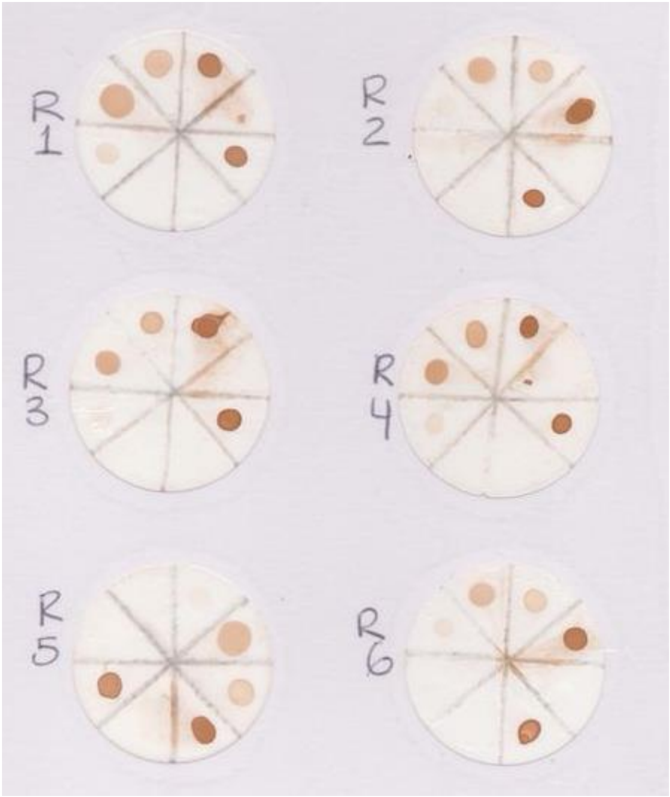

Fig S4. Dot blotting to test the recognition of the peptides by the immunized mice.
