# Supplementary material for: Immunized mice naturally process in silico-derived peptides from the nucleocapsid of SARS-CoV-2
Source: BMC Microbiol. 2023 Oct 28;23:319. doi: 10.1186/s12866-023-03076-5 (PMC10612231; doi:10.1186/s12866-023-03076-5)
Supplement: Supplementary file 2 — Supplementary Material 2 [file 12866_2023_3076_MOESM2_ESM.pdf]

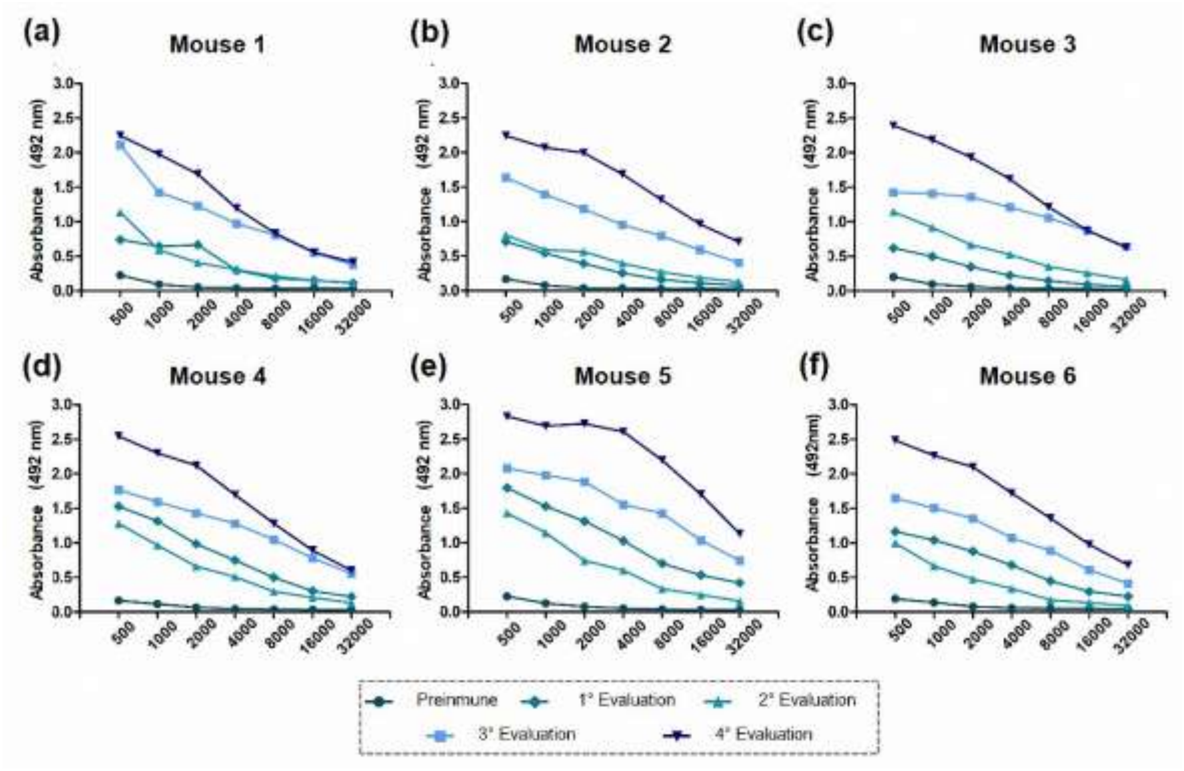


Additional file 1. **Indirect ELISA to test the recognition of the Nrec by the immunized mice.** Sera samples from six mice (a-f) were evaluated at different times: 1, 28, 52, 77, and 127 days. We used antigen Nrec to a final concentration of 1 µg/mL. The primary antibody (serum sample) was diluted from 1:500 to 1:32,000. The secondary antibody was an anti-mice IgG coupled to HRP diluted 1:10,000.
